# Transcriptomic analysis of *Spodoptera frugiperda* Sf9 cells resistant to *Bacillus thuringiensis* Cry1Ca toxin reveals that extracellular Ca^2+^, Mg^2+^ and production of cAMP are involved in toxicity

**DOI:** 10.1242/bio.037085

**Published:** 2019-03-29

**Authors:** Claude Castella, David Pauron, Frédérique Hilliou, Van Tran Trang, Nathalie Zucchini-Pascal, Armel Gallet, Pierre Barbero

**Affiliations:** Université Côte d'Azur, INRA, CNRS, ISA, France

**Keywords:** *Bacillus thuringiensis*, Cry1Ca, Sf9 cells, Resistance, Toxins, Transcriptomic

## Abstract

*Bacillus thuringiensis* (Bt) produces pore forming toxins that have been used for pest control in agriculture for many years. However, their molecular and cellular mode of action is still unclear. While a first model – referred to as the pore forming model – is the most widely accepted scenario, a second model proposed that toxins could trigger an Mg^2+^-dependent intracellular signalling pathway leading to cell death. Although Cry1Ca has been shown to form ionic pores in the plasma membrane leading to cell swelling and death, we investigated the existence of other cellular or molecular events involved in Cry1Ca toxicity. The Sf9 insect cell line, derived from *Spodoptera frugiperda*, is highly and specifically sensitive to Cry1Ca. Through a selection program we developed various levels of laboratory-evolved Cry1Ca-resistant Sf9 cell lines. Using a specific *S. frugiperda* microarray we performed a comparative transcriptomic analysis between sensitive and resistant cells and revealed genes differentially expressed in resistant cells and related to cation-dependent signalling pathways. Ion chelators protected sensitive cells from Cry1Ca toxicity suggesting the necessity of both Ca^2+^ and/or Mg^2+^ for toxin action. Selected cells were highly resistant to Cry1Ca while toxin binding onto their plasma membrane was not affected. This suggested a resistance mechanism different from the classical ‘loss of toxin binding’. We observed a correlation between Cry1Ca cytotoxicity and the increase of intracellular cAMP levels. Indeed, Sf9 sensitive cells produced high levels of cAMP upon toxin stimulation, while Sf9 resistant cells were unable to increase their intracellular cAMP. Together, these results provide new information about the mechanism of Cry1Ca toxicity and clues to potential resistance factors yet to discover.

## Abstract (precis)

**Summary:** Insect cell lines sensitive and resistant to entomopathogenic toxins were developed. Comparative transcriptomic analysis performed on these cell lines provides new information about mechanisms of toxins toxicity.

## INTRODUCTION

*Bacillus thuringiensis* (Bt) is a Gram-positive bacterium that produces proteins with a wide variety of insecticidal properties. These microbial insecticides have been used for decades as pest control agents and they represent an alternative to chemical pesticides in a modern agriculture that strives to be more respectful to the environment and to human health. Moreover, observations of insect resistance to classical chemical pesticides favoured the development and use of the insecticidal weapons produced by Bt (Chattopadhyay and Banerjee, 2018).

The major insecticidal weapons of Bt are two multigenic families of toxins, *cry* and *cyt* (Crickmore et al., 1998). Cry proteins are produced as protoxins in crystal inclusions during Bt sporulation phase. They belong to the pore forming toxins (PFT) class of bacterial toxins (Palma et al., 2014). After spore and crystal ingestion they are delivered to the insect intestinal tract where their activation occurs allowing binding to midgut epithelial cells that results in cell lysis and death of the target insect (Raymond et al., 2010). Two different modes of action on intestinal cells have been proposed and particularly well documented for Cry1A toxins. The first and well-established model, referred to as the pore-forming model, requires the sequential binding to two specific receptors localized at the plasma membrane of insect intestinal cells: a cadherin receptor protein (CADR) and a glycosyl-phosphatidylinositol (GPI) membrane-anchored aminopeptidase N (APN). This sequential binding allows pre-pore complex formation and membrane insertion where they act as functional cationic-specific pores causing osmolytic lysis of targeted cells (Jiménez-Juárez et al., 2007; Soberón et al., 2000; Zhuang et al., 2002). The second model of Cry action, completely independent of pore formation, is referred to as the signal transduction model. Zhang and colleagues showed that an Mg^2+^-dependent signalling pathway is essential to Cry1A-induced cell death. This model also starts with the binding of Cry1A to the primary receptor CADR triggering the recruitment and activation of a heterotrimeric G protein, activation of an adenylyl cyclase (AC), and elevation of intracellular cyclic AMP (cAMP_i_). This second messenger then activates a protein kinase A (PKA) whose activity is shown to be important for toxin-induced cell death (Zhang et al., 2005, 2006). If CADR and APN were the first proteins identified as Cry receptors in insects, numerous other molecules that specifically bind Cry toxins, such as alkaline phosphatase or ABC transporter have been reported (Heckel, 2012; Pigott and Ellar, 2007). The existence of these many potential receptors makes it more difficult to demonstrate a single mode of action of Cry toxins.

Despite all the studies published on Cry1A toxins, numerous events are still missing in the scenario of toxin action leading to insect cell death (Vachon et al., 2012). Cry1C has been described as a pore forming toxin able to oligomerize and form ionic channels after membrane insertion (Laflamme et al., 2008; Peyronnet et al., 2001). Previous studies using histological sections or purified plasma membranes of insect epithelial midgut cells revealed specific Cry1C receptors with low or no competition with Cry1A toxins (Agrawal et al., 2002; Alcantara et al., 2004; Kwa et al., 1998). Cry1C and Cry1A toxins specifically bind to distinct isoforms of APN present in the brush border membrane of *Manduca sexta* insect (Luo et al., 1996; Masson et al., 1995). Moreover, Liu and colleagues have shown that resistance of the diamondback moth to Cry1C was not the result of reduced binding of this toxin to insect midgut membranes, i.e. a resistance mechanism different from that observed for Cry1A-resistant insect (Liu et al., 2000). Finally, Cry1C has been shown to be effective against Cry1A-resistant insects or to act synergistically with Cry1A on target insects (Abdullah et al., 2009; Xue et al., 2005). Pyramiding of the *cry1A* and *cry1C* genes in transgenic plants has been shown to be an effective strategy for delaying the evolution of insect (Tang et al., 2017; Cao et al., 2002). However, field population of diamondback moth repeatedly selected in laboratory for their resistance to Cry1C presented various levels of cross-resistance with members of the Cry1A toxin sub-class (Liu et al., 2001). Taken together, all these data are revealing the complexity of Cry toxin action and are suggesting that Cry1A and Cry1C toxins may have different mechanisms of toxicity. Knowledge of the molecular events involved in Cry1C mode of action would allow a better use of this toxin as a potential alternative to Cry1A and the understanding of resistance mechanisms developed by insects.

The fall armyworm, *Spodoptera frugiperda* (J. E. Smith), is one of the most serious pests ravaging economically important crops across the American continent and was recently spotted in Africa (Goergen et al., 2016; Nagoshi and Meagher, 2008). Transgenic crops expressing Bt toxins have been widely and efficiently used against *S. frugiperda*. However, numerous cases of laboratory- or field-evolved resistance to Cry toxin have been reported (Farias et al., 2014; Storer et al., 2010). In order to mimic strategies developed by *S. frugiperda* to reduce its susceptibility to toxins we engineered a resistant cellular model using Sf9 cells. These cells are derived from *S. frugiperda* and have been intensively used as an insect model to study Cry1C toxicity. Among all the Lepidoptera cell lines studied by Kwa and colleagues, Sf9 cells appeared highly and specifically sensitive to Cry1C with more than 98% of the cells affected (swollen cells) after 3 h of toxin treatment (Kwa et al., 1998). However, a cell cycle-dependent resistance to Cry1C was observed in Sf9 cells. Cells arrested in G2-M phase were insensitive to Cry1C and gradually regained their sensitivity when the arresting agent was removed (Avisar et al., 2005).

In the present study we selected two levels of resistance to Cry1Ca in Sf9 cells. Genes differentially expressed between resistant selected cell lines and the wild-type sensitive one were studied. Our analysis led us to focus on the molecular mechanism involving cation-dependent cellular pathway that could be involved in Cry1Ca toxicity.

## RESULTS

### Development and characterization of Sf9 cells resistant to Cry1Ca toxin

**Fig. 1.**
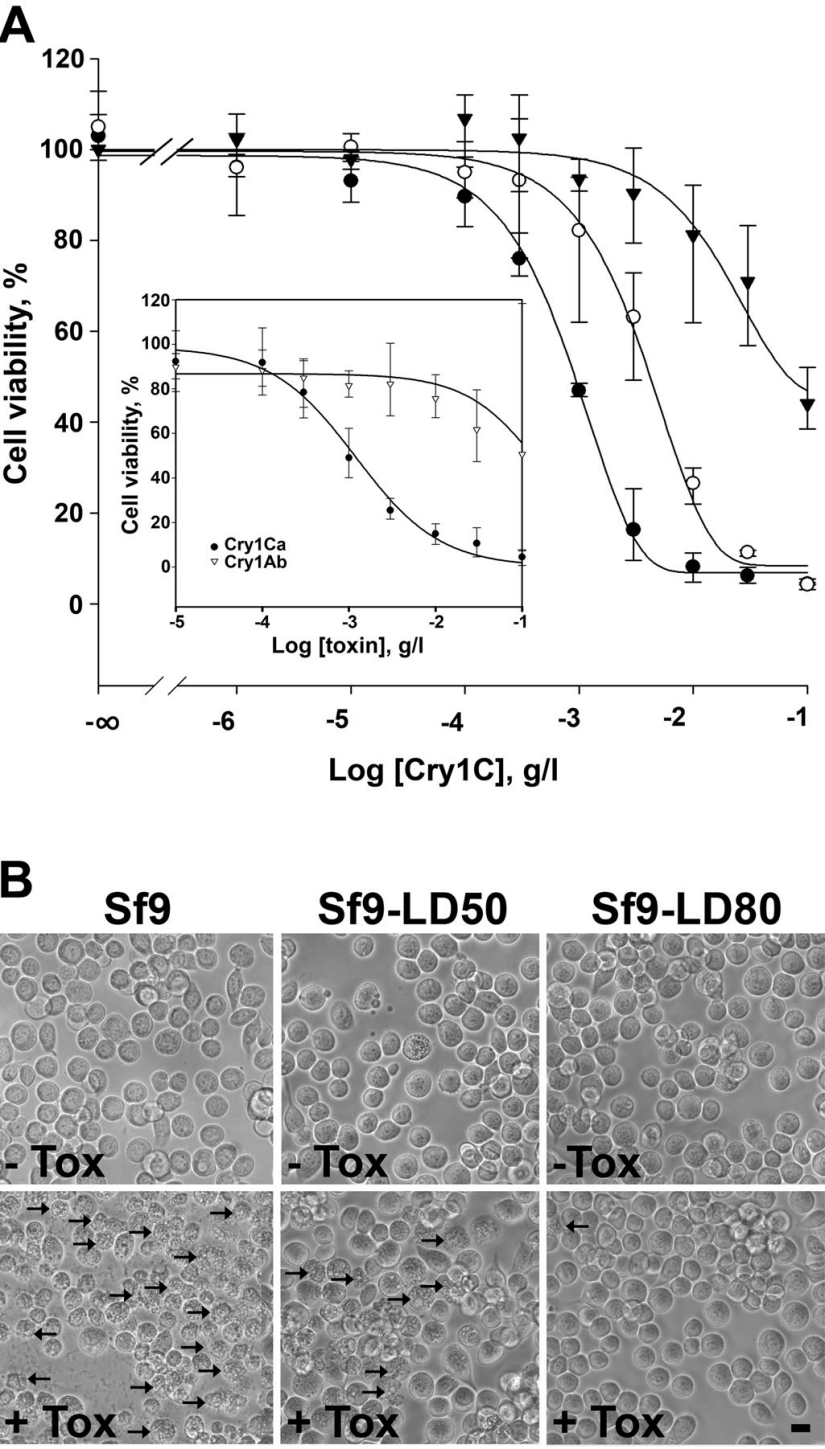
**Sensitivity of Sf9wt cells to Cry toxins and development of populations resistant to Cry1Ca.** (A) Cells were incubated in complete media containing various concentrations of Bt toxins. Cell viability was evaluated by SYBR Gold staining. Inset shows the sensitivity of Sf9wt cells to Cry1Ca toxin (●) as compared to Cry1Ab toxin (∇). Main graph shows cell viability measurements of selected resistant Sf9 cell lines Sf9-LD_50_ (○) and Sf9-LD_80_ (▾) compared to unselected Sf9wt (●) in the presence of Cry1C toxin. Data are presented as the mean±s.d. of three independent experiments performed in duplicate. Cry1Ca lethal concentration giving 50% dead cells (LD_50_) is deduced from the curves. (B) Sensitive or resistant Sf9 cell lines were incubated for 18 h in toxin-free media (-Tox) or containing 0.9 mg/l of Cry1Ca toxin (+Tox) and observed using phase contrast microscopy. Shown results are those from one experiment representative of three independent experiments. Black arrows point to examples of affected cells. Scale bar: 20 µm.

Sf9 cells are known to be susceptible to the Cry1C Bt toxin (Kwa et al., 1998). We first verified if the Sf9 cell line we were using was still highly and specifically sensitive to the Cry1Ca toxin. Viability tests were performed onto Sf9 wild-type (Sf9wt) cells in the presence of increasing concentrations of different Bt toxins (Fig. 1A). Solubility problems prevented us from calculating LD_50_ of Cry1Ab on Sf9 cells. Nevertheless, as observed in the inset in Fig. 1 and as reported by others (Bretschneider et al., 2016) Cry1Ab (white triangles) was not efficient in killing cells, while Cry1Ca (black circles) efficiently killed the cells with a lethal dose, giving 50% dead cells (LD_50_) evaluated at 0.9±0.15 mg/l. In order to study the mode of action of Cry1Ca and to investigate resistance mechanisms observed in *S. frugiperda*, we started a selection program to develop cell strains resistant to this toxin. Doses calculated for Sf9wt cells and corresponding to the LD_50_ and LD_80_ were used to select cells over 40 generations. At the end of the selection program, resistant cell lines obtained using LD_50_ or LD_80_ doses were named Sf9-LD_50_ and Sf9-LD_80_ respectively. Cell viability was tested at the 40th generation and LD_50_ measured for Sf9-LD_50_ (white circles in Fig. 1A) and Sf9-LD_80_ resistant cells (black triangles in Fig. 1A) were 4.4±1.6 mg/l and 53±13 mg/l, respectively. We calculated resistance factors (RF) using LD_50Sf9-resistant_/LD_50Sf9wt_ ratio: RF=4.9±0.8 for Sf9-LD_50_ cells and RF=58.9±2.8 for Sf9-LD_80_ cells. These resistant cells presented no significant time growth difference as compared to Sf9wt cells used for the selection program (data not shown). Cells were incubated for 18 h with 0.9 mg/l of Cry1Ca and observed using phase contrast microscopy (Fig. 1B). Both untreated Sf9 resistant cells presented no significant morphological difference compared to Sf9 sensitive cells without toxin in the media. When Cry1Ca was added in the media, Sf9wt cells showed morphological changes with vacuole formation and lysis. While a large number of Sf9wt cells were killed, only a few Sf9-LD_50_ cells were affected and no change in Sf9-LD_80_ cell density and morphology was observed.

### Cry1Ca still binds to the plasma membrane of resistant Sf9 cell lines

**Fig. 2.**
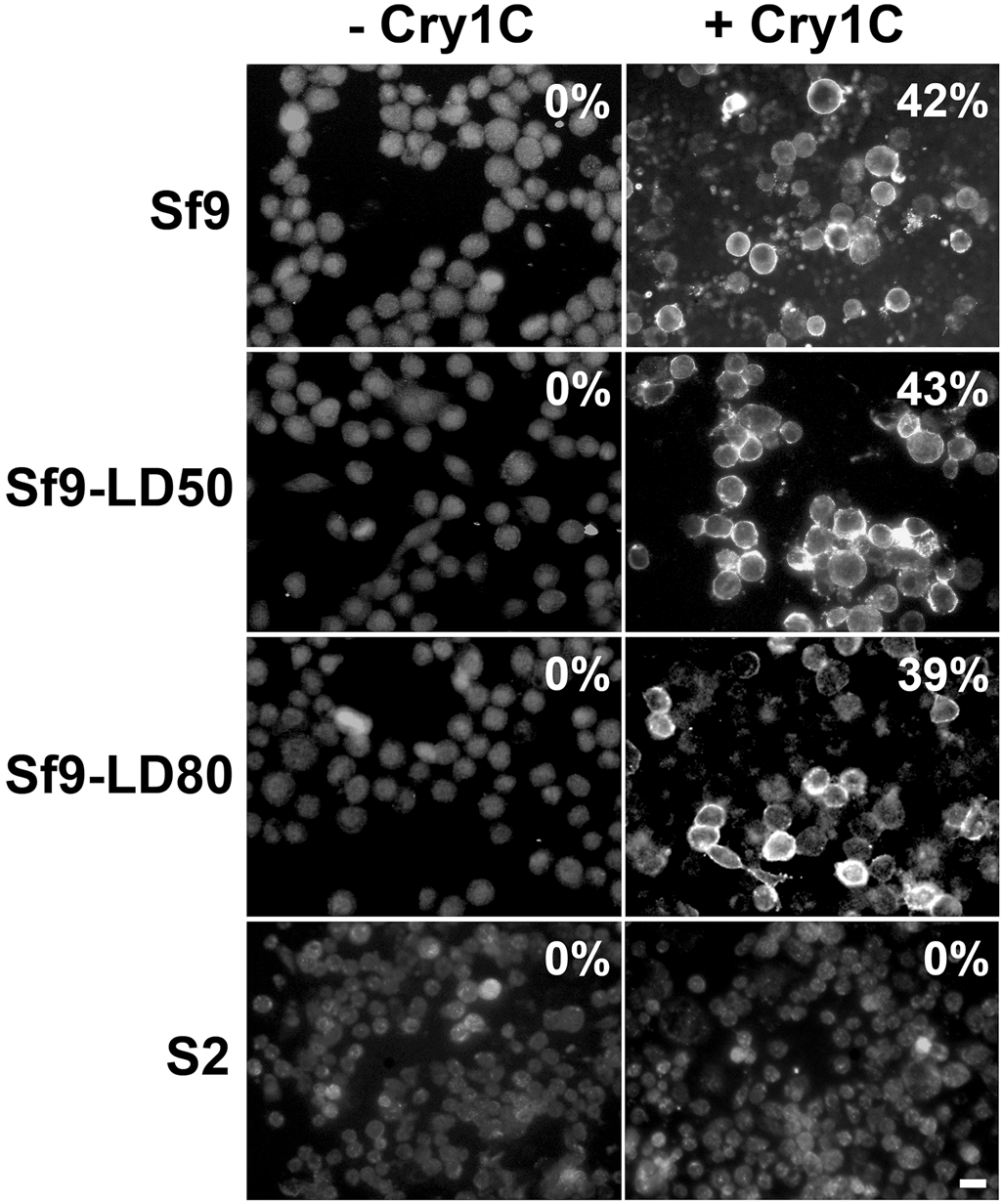
**Cry1Ca binding to the plasma membrane of Sf9 and S2 cell lines,** **Sf9wt cells or resistant cell lines Sf9-LD_50_ or Sf9-LD_80_ were incubated 40 min** **in complete media free of toxin (−Cry1C) or containing 3 mg/l of Cry1Ca toxin (+Cry1C).** Immunostaining was performed using primary antibodies to Cry1Ca and secondary antibodies coupled to Alexa Fluor 488. Toxin localization was visualized using an inverted epifluorescence microscope. As a control, immunostaining was carried out onto cells incubated without toxin (−Cry1C) and S2 cells (S2+Cry1C) insensitive to Cry1Ca toxin. The numbers indicate the percentage of cells immunostained. Shown results are those from one experiment representative of two independent experiments. Scale bar: 20 µm.

Among all known resistance mechanisms to *Bacillus* entomopathogenic toxins, so far loss of plasma membrane receptor binding appears to be one of the major mechanisms developed by insects (Heckel et al., 2007). In order to verify if our resistant cell lines established such a resistant mechanism, sensitive or resistant cells were incubated for 40 min with complete media containing 3 mg/l of Cry1Ca and immunostaining of the toxin was performed (Fig. 2). We found no detectable difference in Cry1Ca membrane binding between sensitive and resistant cells. When compared to the control where no toxin was present in the media (left row), sensitive and resistant cell lines showed comparable numbers of cells stained by antibodies to Cry1Ca. Most of the plasma membranes were uniformly labelled with 2few punctuates eventually caused by toxin clustering and/or oligomerization. The same experiment was performed with *Drosophila Schneider 2* (S2) cells known to be insensitive to Cry1Ca toxin (bottom row). We found no Cry1Ca signal associated with the plasma membrane of S2 cells supporting the idea that toxin membrane binding is necessary for toxin action. Our data suggested that the observed resistance in Sf9-LD_50_ and Sf9-LD_80_ cells is probably not caused by loss of a plasma membrane receptor.

### Comparative transcriptomic analysis of Sf9 resistant versus sensitive cell lines

**Fig. 3.**
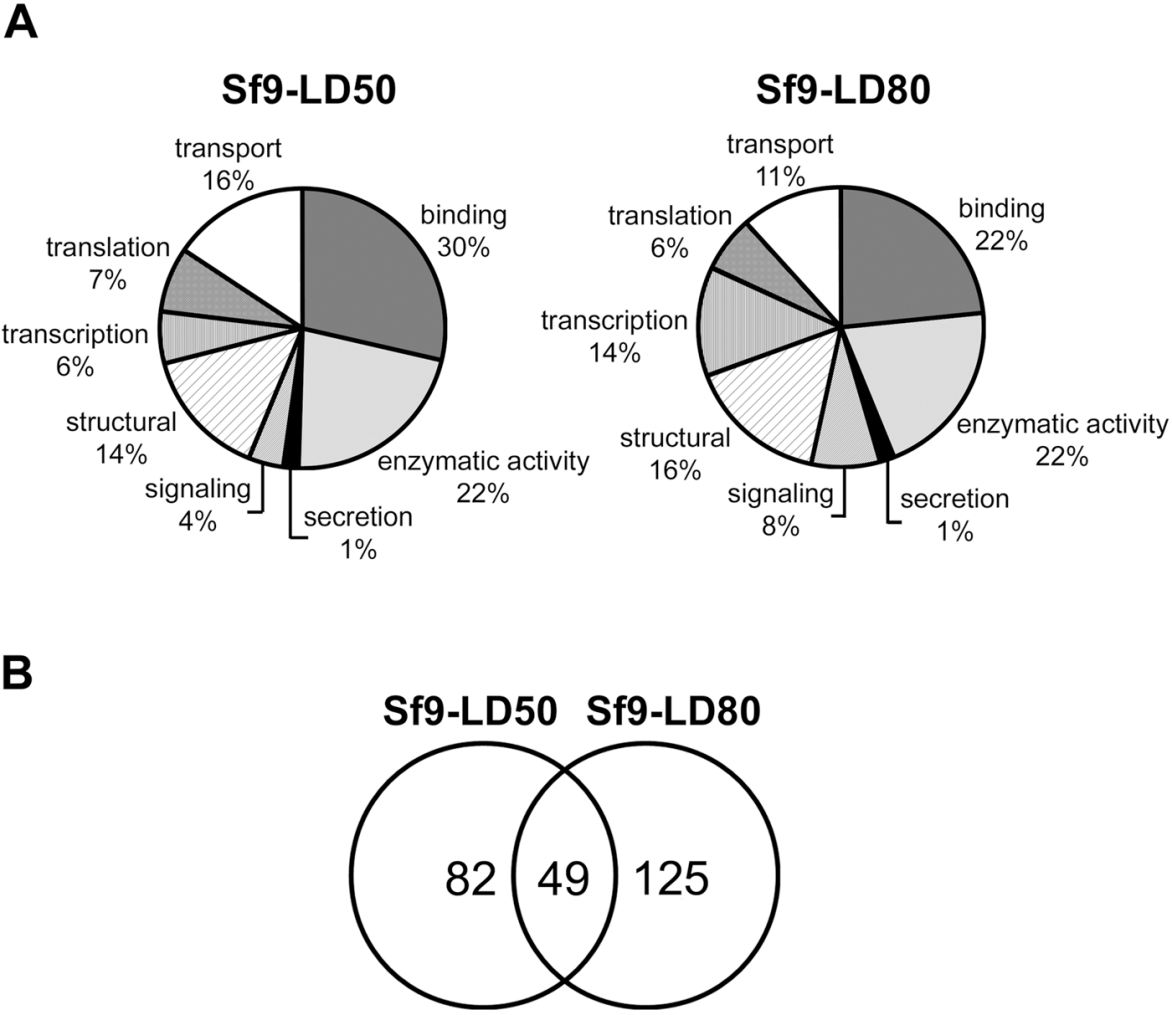
**Transcriptomic analysis of resistant Sf9 cell lines.** (A) Pie charts of the biological functions of detected genes over or under-expressed in Sf9 resistant cell lines. Sf9-LD_50_ and Sf9-LD_80_ are compared to Sf9wt sensitive cells. Genes identified on the Spodo Database are classified into seven categories using Gene Ontology terms: transport, binding, enzymatic activity, secretion, signalling, structural, transcription and translation. (B) Venn diagram showing number of genes differentially expressed in Sf9-LD_50_ only (82), in Sf9-LD_80_ only (125) and common in both resistant cell lines (49).

Comparative transcriptomic analysis was performed onto total mRNA isolated from sensitive Sf9 cells and resistant selected cells. Expression levels were analysed on a specific *S. frugiperda* microarray representing 9721 genes. To evaluate the potential effects of cell aging and culture condition on gene expression, we compared the expression patterns of the 8th (Sf9p8) and 40th (Sf9p40) generations of wild-type cells. Only three showed genes-coding for a Rho GTPase, a fatty acid-binding protein 2 (FABP2) and an unidentified protein among the 9721 tested genes that were significantly differently transcribed (Table S1, sheet 1). The variations observed for the Rho GTPase and the FABP2 are in accordance with previous studies showing that mRNA and protein levels of these two molecules are regulated throughout the cell cycle and cell aging, and are involved in the regulation of key components of the cell cycle (Coleman et al., 2004; Pu et al., 1999; Yoon et al., 2007). The low gene expression variation observed between Sf9p8 and Sf9p40 validated our global approach to identify genes with expression profiles specifically modified during the establishment of Cry1Ca resistance in Sf9 cells. Classification of the differentially expressed genes using Gene Ontology terms revealed numerous biological processes modified in both resistant selected cells: transcription, translation, transport, binding, enzymatic activity, secretion, signalling and structural (Fig. 3A). Among all these biological processes, we observed that as RF increases, an enrichment of terms related to the cell signalling pathway (from 4% in Sf9-LD_50_ to 8% in Sf9-LD_80_) and to transcription processes (from 6% in Sf9-LD_50_ to 14% in Sf9-LD_80_) are seen. Moreover, as shown in the Venn diagram proposed in Fig. 3B, while 82 and 125 genes presented variation of expression specifically in Sf9-LD_50_ and in Sf9-LD_80_ cells respectively, only 49 genes appeared to be common to both selected cell lines. Thirty-six out of these 49 common genes differentially up- or downregulated in the same way in both resistant cell lines and were identified (Table 1). While numerous cellular processes were modified, we thought that these common genes could play a central role in the mechanism of resistance to Cry1Ca or in its mode of action. Among these 36 identified common genes, 17% appeared to be possibly involved in cellular pathways requiring or modifying cation intracellular concentrations. Moreover, Omic analysis revealed that cation homeostasis and/or cation-dependent cell signalling processes were modified in cells or insects resistant to Bt toxins (Candas et al., 2003; Canton et al., 2015; Nanoth Vellichirammal et al., 2015). All these observations led us to investigate the importance of cations in Cry1Ca toxicity using Sf9-LD_80_ only; the selected cell lines with the highest calculated RF.

**Table 1.**
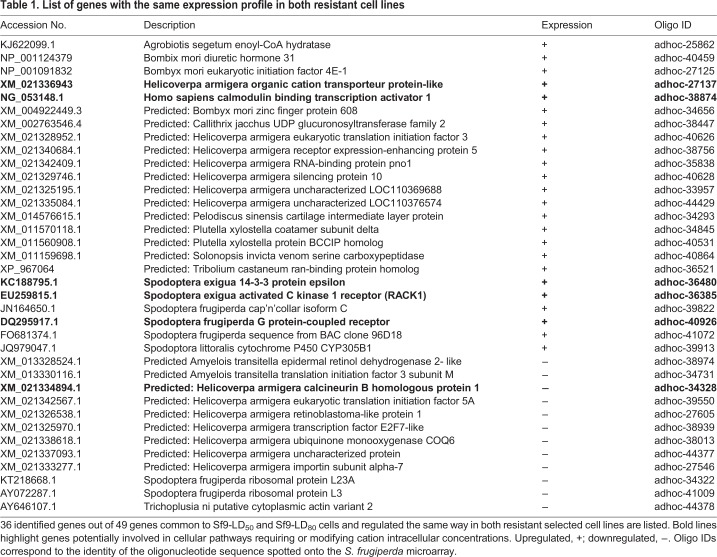
**List of genes with the same expression profile in both resistant cell lines**

### Protective effect of divalent ions chelators

**Fig. 4.**
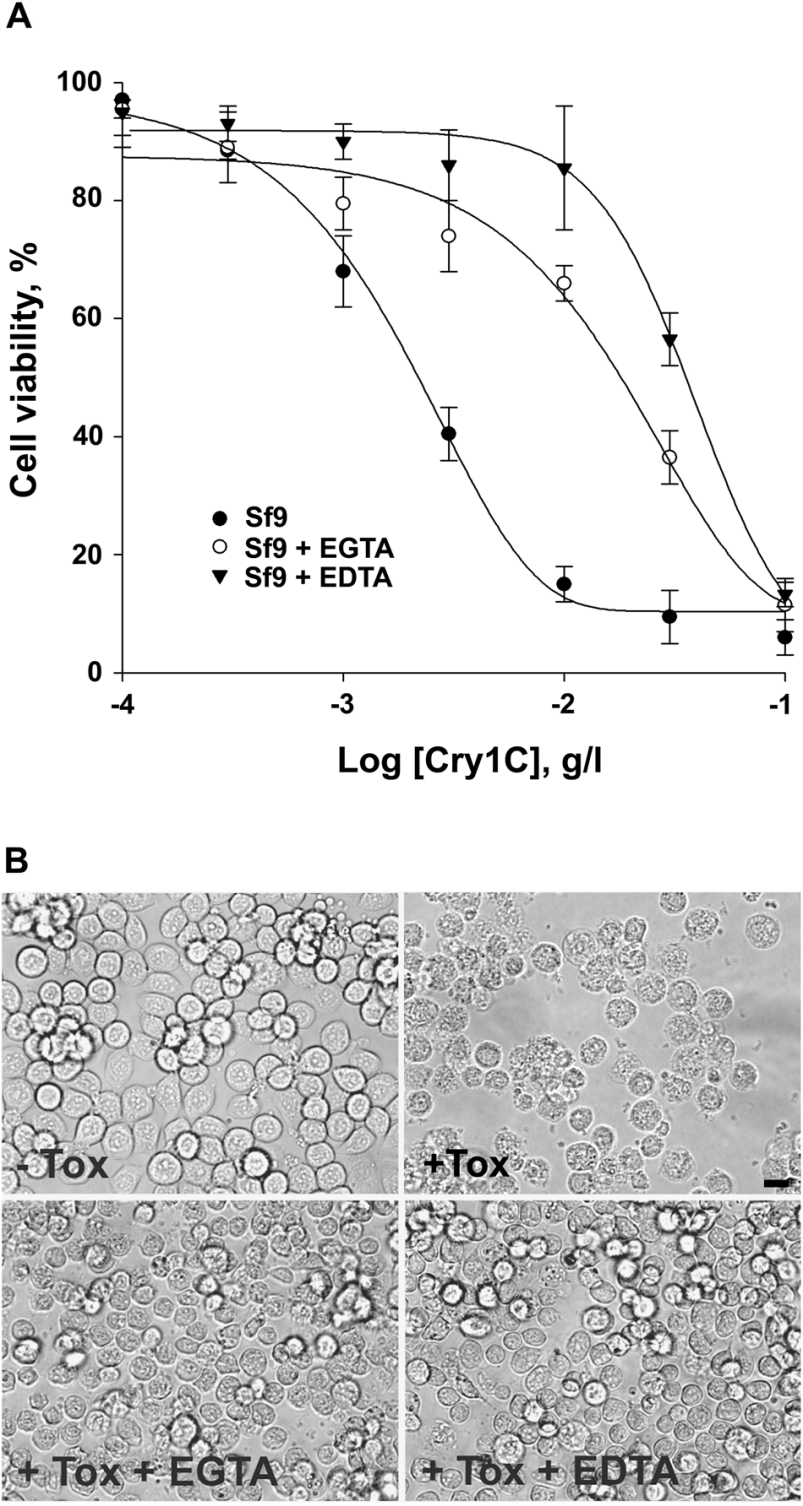
**Sf9wt cells are protected from Cry1Ca toxicity by ion chelators.** (A) Sf9wt cells were incubated for 18 h in complete media containing various concentrations of Cry1Ca toxin alone, Cry1Ca toxin +5 mM EGTA or Cry1Ca toxin +5 mM EDTA. Cell viability was evaluated by SYBR Gold staining. Data are presented as the mean±s.d. of three independent experiments done in duplicate. (B) Sf9wt cells were observed using phase contrast microscopy 18 h after incubation with 5 mg/l of Cry1Ca toxin (+Tox), Cry1Ca toxin +5 mM EDTA (+Tox +EDTA) or Cry1Ca toxin +5 mM EGTA (+Tox +EGTA). Cell morphology was compared to Sf9wt cells in complete media only (−Tox). Shown results are those from one experiment representative of three independent experiments. Scale bar: 20 µm.

**Fig. 5.**
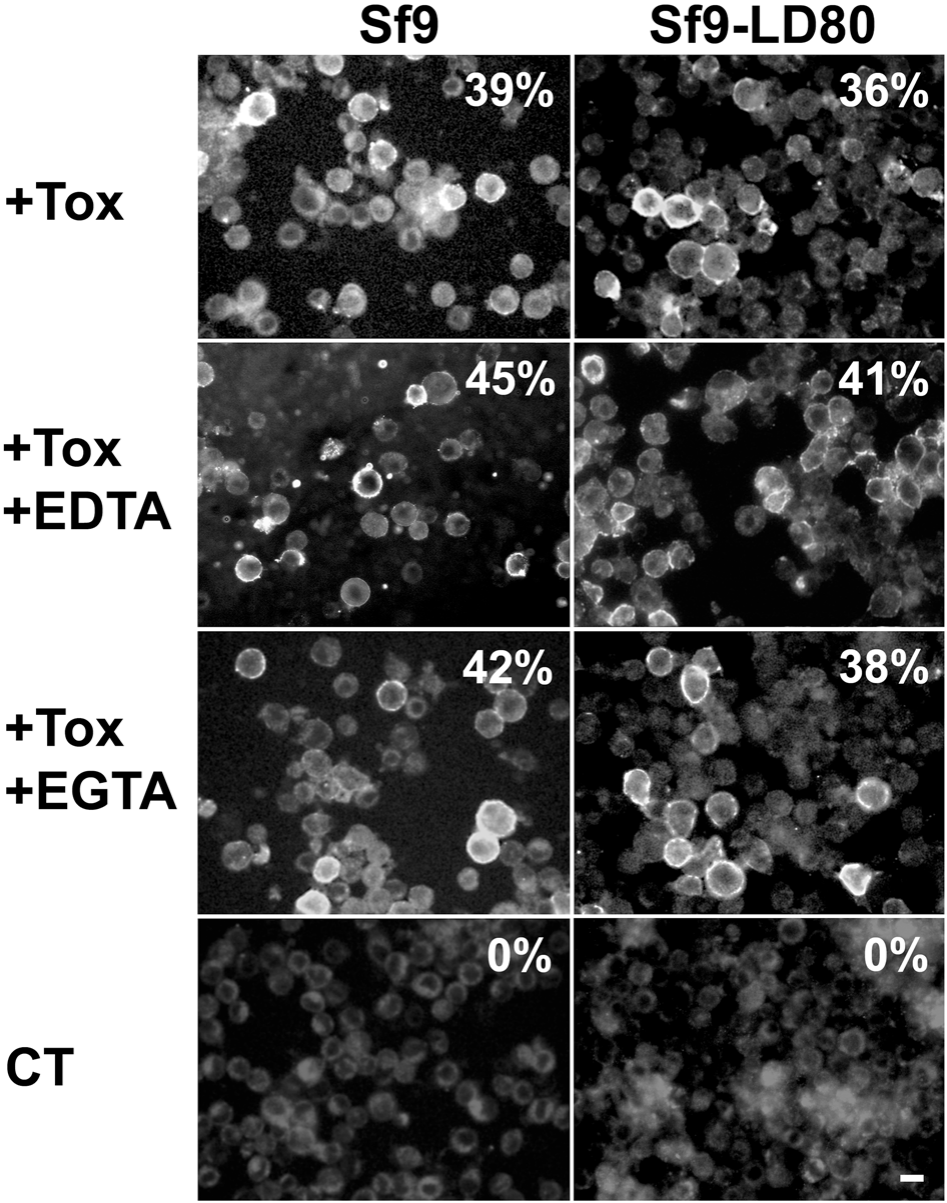
**Cry1Ca plasma membrane binding is not affected by the presence of chelators.** Sf9wt cells (left row) or Sf9-LD_80_ cells (right row) were incubated for 40 min in complete media containing 3 mg/l of Cry1Ca toxin alone (+Tox), Cry1Ca toxin +5 mM EDTA (+Tox +EDTA) or Cry1Ca toxin +5 mM EGTA (+Tox +EGTA). Immunostaining was performed using primary antibodies to Cry1Ca and secondary antibodies coupled to Alexa Fluor 488. Toxin localization was visualized using an inverted epifluorescence microscope. As a control (CT), immunostaining was carried out onto cells incubated without toxin. The numbers indicate the percentage of cells immunostained. Shown results are those from one experiment representative of two independent experiments. Scale bar: 20 µm.

Divalent ions have been described as common key players in the main proposed modes of action of Bt toxins (Zhang et al., 2006). To investigate their contribution to Cry1Ca toxicity, we carried out Sf9 cells intoxication during 18 h with different concentrations of toxin in the presence of 5 mM EDTA or 5 mM EGTA and we evaluated cell viability. EDTA chelates Mg^2+^ and Ca^2+^, whereas EGTA preferentially chelates Ca^2+^. No detectable mortality was observed when Sf9wt or Sf9-LD_80_ cells were incubated with chelators only (data not shown). As shown in Fig. 4A, both EDTA and EGTA displayed a protective effect regarding toxin action when compared to Sf9 cells intoxicated without ion chelators in the media. The LD_50_s measured for Sf9wt (black circles), Sf9wt with EDTA (black triangles) and Sf9wt with EGTA (white circles) were 2.1±0.4, 35.8±6 and 18±5 mg/l of Cry1Ca, respectively. Calculation of LD_50_s ratios revealed an 8.57±0.76-fold decrease in Cry1Ca toxicity when EGTA was present in the media and a 17.05±0.43-fold decrease for EDTA. Observation under a light microscope revealed a slight effect on the morphology of Sf9wt protected cells (Fig. 4B) and Sf9-LD_80_ resistant cells (data not shown). Most of the Sf9wt cells protected by chelator presence (+Tox +EDTA or +Tox +EGTA) were still intact compared to cells with Cry1Ca only (+Tox), but they appeared less flattened and round shaped than untreated Sf9 (−Tox). These minor morphological changes were not observed when both cell lines were incubated with media containing chelators only (data not shown) suggesting that these changes were not the result of cell adhesion alteration caused by extracellular ions clustering. In order to verify whether the observed protective effect of both EDTA and EGTA was not the result of Cry1Ca binding loss onto Sf9 plasma membrane, we performed immunostaining using Cry1Ca primary antibodies in presence of 5 mM chelators (Fig. 5). Control cells (CT) were incubated with EDTA only. The percentage of membrane stained cells was calculated in the different conditions. While 0% of total cells were membrane-labelled in the control, we did not observe significant difference in toxin binding onto Sf9wt membranes in presence or absence of EDTA or EGTA. Once again, Sf9-LD_80_ cells presented comparable results to Sf9wt cells with about one-third of total cells positive for toxin binding. All these results revealed a significant protective effect of cationic chelators suggesting an important contribution of both Mg^2+^ and Ca^2+^ in Cry1Ca action. This protective effect was not likely due to loss of interaction of Cry1Ca with plasma membrane binding regions.

### Cry1Ca toxin induces Cyclic AMP production

**Fig. 6.**
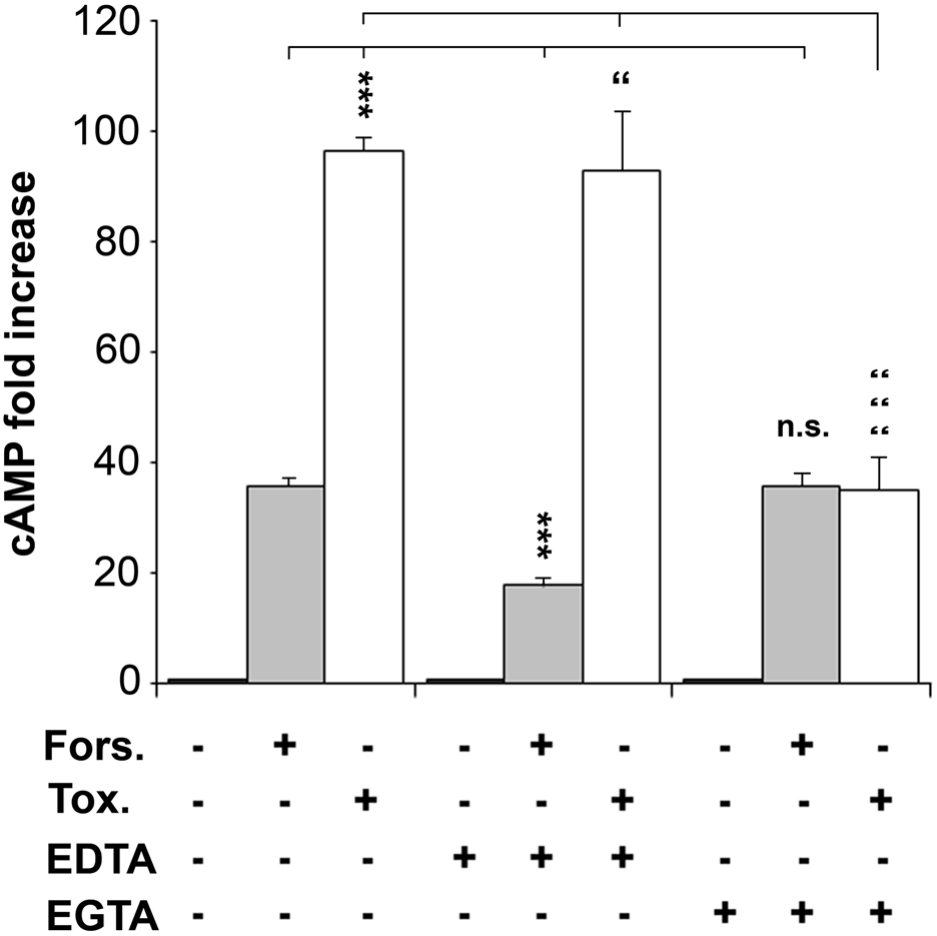
**cAMP is produced in Sf9wt cells after toxin treatment.** Sf9wt cells were incubated 20 min in complete media only (−) or media supplemented with (+) 100 µM forskolin (Fors.), 1 mg/l Cry1Ca toxin (Tox.), 5 mM EDTA or 5 mM EGTA. After incubation, cytosolic HCl extract were performed and cAMP amount measured using an immunoassay kit. cAMP fold increase was evaluated compared to non-stimulated cells (see Materials and Methods). Data are presented as the mean±s.e. of three independent experiments done in duplicate. Student's *t*-tests were used for comparisons among groups. ***P<0.001; n.s., not significant compared to the condition with forskolin only. “*P*<0.05, “““*P*<0.001, compared to the condition with toxin only.

Previous studies proposed that Cry protein toxicity involves activation of an Mg^2+^-dependent signalling pathway leading to the production of the second messenger cAMP (Zhang et al., 2006). Since (i) divalent ions chelators appeared to have a protective effect upon Cry1Ca toxicity and (ii) numerous crosstalk have been described between cAMP pathway and calcium signal (Hofer, 2012), we incubated Sf9 cells with the toxin in media with or without chelators and we evaluated cAMP production (Fig. 6). Activation with forskolin, a direct adenylate cyclase (AC) activator, was used as a positive control for cAMP production. As expected, Sf9wt cells treated with 100 µM forskolin presented a 35-fold increase of cAMP level when compared to untreated cells. A significantly stronger effect was observed in Sf9wt cells treated with Cry1Ca, with a 96-fold increase. When stimulations were achieved in presence of EDTA in the media, we observed a specific inhibitory effect on cAMP production affecting forskolin treatment while cAMP production triggered by Cry1Ca was only slightly affected. Indeed, the fold increase in cAMP using forskolin or Cry1Ca were respectively reduced to 18-fold and 92-fold. Finally, when EGTA was present in the media of stimulated cells we observed a specific inhibitory effect onto cAMP production due to Cry1Ca toxin with a cAMP fold increase significantly reduced from 96-fold to 35-fold. We observed no EGTA inhibitory effect on cAMP levels obtained after forskolin treatment. Taken together these results showed that Cry1Ca was able to trigger the production of cAMP in Sf9 cells through a mechanism requiring extracellular Ca^2+^.

### Sf9-LD_80_ cells are impaired in cAMP production

**Fig. 7.**
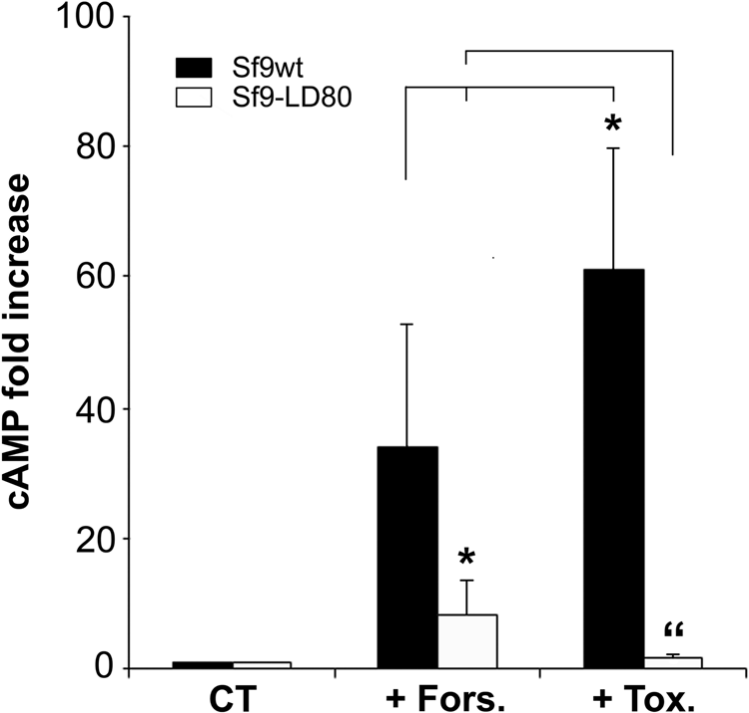
**Sf9-DL80 cells are affected in cAMP production.** Sf9wt or Sf9-LD_80_ cells were incubated for 20 min in complete media only (CT) or media supplemented with 100 µM forskolin (+Fors.) or 1 mg/l Cry1Ca toxin (+Tox.). After incubation, cytosolic HCl extracts were performed and cAMP amount measured using an immunoassay kit. cAMP fold increase was evaluated compared to non-stimulated cells (see Materials and Methods). Data are presented as the mean±s.e. of three independent experiments done in duplicate. Student's *t*-tests were used for comparisons among groups. *P<0.05, compared to Sf9wt cells stimulated with forskolin only. “*P*<0.05, compared to Sf9-LD_80_ stimulated with toxin only.

Since Cry1Ca action seemed to require increase of intracellular concentration of cAMP ([cAMP]_i_) we wondered whether the resistant cell line Sf9-LD_80_ was able to produce this second messenger. Sf9wt sensitive cells or Sf9-LD_80_ resistant cells were stimulated with forskolin or Cry1Ca and the increase in cAMP was measured (Fig. 7). In contrast to what we observed for Sf9wt cells, Sf9-LD_80_ had an altered response to stimulation by both forskolin and the toxin. While 100 µM forskolin increased the cAMP amount eightfold (less than one quarter of what we observed for Sf9wt cells), no cAMP increase was detected after stimulation with Cry1Ca. This experiment suggests that Sf9-LD_80_ cells could overcome Cry1Ca toxicity by blocking cAMP-dependent signalling normally triggered by the toxin in sensitive cells.

## DISCUSSION

In this report, we describe Sf9 cells resistant to the Cry1Ca toxin obtained after a selection program. We tested Sf9wt cells for their sensitivity to another Cry toxin (Cry1Ab) at toxin concentrations up to 100 mg/l in the same time conditions as with Cry1Ca and confirmed that they were almost ineffective on these cells, suggesting a peculiar mode of action for Cry1Ca. The LD_50_ measured with these cells that are specifically sensitive to Cry1Ca were in the same concentration range to what was observed in previous studies (Kwa et al., 1998). Therefore, we started our selection program using LD_50_ or LD_80_ Cry1Ca doses and we obtained cell lines with an increasing resistance factor, named Sf9-LD_50_ and Sf9-LD_80_.

While the so-called ‘mode 1 resistance’, the most frequently mechanism of Cry toxins resistance, results in toxin receptor binding defects (Gahan et al., 2001; Jurat-Fuentes et al., 2011; Morin et al., 2003) we observed no detectable difference in Cry1Ca binding to the plasma membrane of Sf9 sensitive or resistant cells (Fig. 2). It is important to note that this approach using immuno-fluorescence microscopy could not rule out an undetectable small diminution of receptor affinity or loss of a secondary receptor, which could be sufficient to perturb toxin toxicity onto Sf9-LD_80_ cells. However, our binding results are strongly correlated with our transcriptomic analysis. Indeed, we did not detect any significant variation of expression of previously described and widely accepted plasma membrane toxin receptor as CADR or APN. All these results strongly suggest that Cry1Ca resistance observed in the Sf9-LD_50_ or Sf9-LD_80_ cells is probably not caused by loss or alteration of plasma membrane binding.

Use of differential transcriptomic analysis to understand resistance mechanisms developed by insects to counteract toxicity is a challenging task. It generates a large amount of data and shows that cellular responses are likely diverse and affect many cellular processes as observed in Fig. 3A and in other studies (Nanoth Vellichirammal et al., 2015; Canton et al., 2015). Therefore, we decided to focus on genes differentially expressed in the same way in both selected resistant cells, as they may be key players in resistance processes (Table 1). Hence, we highlighted genes involved in cation-dependent signalling pathways or cation homeostasis. For example, calmodulin-binding transcription activator (CAMTA) is activated after a rise in intracellular calcium concentration ([Ca^2+^]_i_). Both Ca^2+^ and calmodulin (Cam) bind to CAMTA, which translocates into the nucleus and activates its target genes (Finkler et al., 2007). Like CAMTA, the predicted Calcineurin B homologue was differentially expressed in both selected resistant cell lines. Calcineurin is a calcium-activated protein phosphatase that participates in diverse biological functions through interaction with various partners (Li et al., 2011). It is composed of two subunits, a catalytic subunit (CnA) and a regulatory subunit (CnB) containing Ca^2+^ binding sites. Increased intracellular Ca^2+^ allows binding of the cations to the regulatory sites, which initiates a series of conformational changes and the activation of calcineurin (Yang and Klee, 2000). Thus, in both examples a rise in [Ca^2+^]_i_ appears as a crucial event in the activation of our potential key players in Cry1Ca resistance.

Earlier studies showed the involvement of divalent cations in Cry activity. Cry1Ca toxicity increases with the presence of calcium in a dose-dependent manner and the toxin induces a rapid elevation of intracellular calcium in treated cells (Monette et al., 1997). We followed intoxication of Sf9 sensitive cells in the presence of EDTA or EGTA and observed a protective effect for both ion chelators (Fig. 4). Similar to the observations for Cry1Ab binding onto S5 cells (Zhang et al., 2005), neither EDTA nor EGTA affected the interaction of Cry1Ca with the plasma membrane of Sf9wt or Sf9-LD_80_ cell lines (Fig. 5). The protection obtained with the Ca^2+^ chelator EGTA was not the result of a loss of toxin binding capacity to Sf9wt cells and could be the result of the failure of the cells to increase their [Ca^2+^]_i_ in response to the toxin. If pore formation or the signal transduction pathway are the two main models of toxin action leading to cell death, a third model was proposed in which formation of toxin oligomers followed by pore formation due to oligomer insertion in cell membrane could participate in establishment of a signalling pathway (Jurat-Fuentes and Adang, 2006). Pores composed of Cry1Ca toxin do not induce only potassium-selective channels but increase the permeability of the plasma membrane of Sf9 cells to numerous solutes (Guihard et al., 2000; Villalon et al., 1998). The toxin has been shown to trigger an intracellular calcium surge in Sf9 cells and is still toxic when Sf9 cells are incubated in media containing barium, a divalent cation used as potassium channel blocker (Monette et al., 1997; Schwartz et al., 1991). So, the protective effect of calcium chelators could result from blocking Ca^2+^ entry in the cell through poorly selective channels obtained by toxin membrane insertion and the incapacity of this calcium surge to contribute to a Ca^2+^-dependent signal pathway, leading to cell death. When we used the Mg^2+^ chelator EDTA, we observed a stronger decrease of Cry1Ca toxicity compared to protection obtained with EGTA (Fig. 4). Like Zhang and colleagues, who observed that Cry1Ab still binds to the plasma membrane of High five cells in the presence of EDTA (Zhang et al., 2005), we saw no binding loss of Cry1Ca to Sf9wt sensitive or Sf9-LD_80_ resistant cells when incubated with 5 mM EDTA (Fig. 5). So, as described for Cry1Ab, depletion of Mg^2+^ using EDTA could prevent Cry1Ca-induced cellular response and stop the establishment of a Mg^2+^-dependent intracellular pathway critical to promote cell death (Zhang et al., 2006). Since Mg^2+^ is essential for adenylyl cyclase (AC) to catalyse the formation of cAMP (Tesmer et al., 1999; Pieroni et al., 1995) and since AC activity has been shown by Zhang et al. (2005) to participate in Cry1Ab toxicity, we monitored cAMP production after Sf9 cell intoxication with Cry1Ca (Fig. 6). Treatment of Sf9 sensitive cells with forskolin, used as positive control, produced a 38-fold increase of intracellular [cAMP] while Cry1Ca presented an even stronger stimulation of cAMP production. [cAMP]_i_ increase after toxin treatment was not affected by the presence of EDTA, whereas the cAMP increase was reduced two fold in Sf9wt cells stimulated with forskolin plus EDTA as compared to forskolin alone. In contrast, EGTA affected only Cry1Ca-induced cAMP production. These results suggest the importance of Ca^2+^ ions in toxin action via formation of cAMP. Since numerous AC activities have be shown to be regulated by [Ca^2+^]_i_ (Willoughby and Cooper, 2007), Cry1Ca, after formation of pores in the Sf9 membrane, could activate AC through entry in cells of extracellular Ca^2+^. Näsman's work showed that applying 1 mM EGTA to Sf9 cell media blocked the raise in [Ca^2+^]_i_ and drastically reduced the increase of cAMP levels after stimulation with octopamine (Näsman et al., 2002). Thus, the increase in cAMP levels observed in our cells treated with Cry1Ca could be due to the activation of calcium-dependent AC, with EGTA protecting cells by preventing the increase in calcium and AC activation.

If EGTA was the most efficient to reduce cAMP elevation in response to Cry1Ca stimulation, EDTA which preferentially binds Mg^2+^ appeared the most protective against Cry1Ca toxicity. This effect of EDTA could be explained, on one hand, by the necessity of Mg^2+^ for catalytic activity of ACs. Indeed, Mg^2+^ which binds to the active domain of ACs is essential for their activity and therefore for the synthesis of cAMP (Pieroni et al., 1995; Tesmer et al., 1999). On the other hand, Mg^2+^ could have a more central role than Ca^2+^ in participating in the activation of numerous proposed other partner proteins involved in toxin-induced signalling pathway, beginning with heterotrimeric G protein activation (Sprang, 1997). However, while in the absence of Mg^2+^ we observed an effect on cAMP production by forskolin stimulation, EDTA did not seem to prevent the increase of cAMP after the action of the toxin. Cry1Ca might be triggering the production of cAMP by activation of an Mg^2+^-independent AC whose regulation requires Ca^2+^ (Braun et al., 1977; Litvin et al., 2003).

It has been proposed that the mechanism of action of the toxin can be independent of cAMP production (Knowles and Farndale, 1988; Portugal et al., 2017). Portugal and colleagues clearly demonstrated that both Cry1Ab and Cry1Ac toxins triggered cell death in CF1 cells via pore formation activity. While they saw neither the activation of PKA nor an increase in intracellular cAMP concentration during Cry1Ab or Cry1Ac intoxication, in our hands Cry1Ca triggered an increase of the [cAMP]_i_ in favour of a peculiar mode of action of this toxin (Fig. 6). Moreover, we observed (Fig. 7) that the Sf9-LD_80_ resistant cell line was unable to elevate [cAMP]_i_ in response to Cry1Ca treatment. Thus, the [cAMP]_i_ increase seems to be an essential element to the toxicity induced by Cry1Ca but appeared insufficient since forskolin, although it may increase cAMP levels, presents no toxicity onto Sf9 cells. While Zhang and colleagues observed a similar lack of forskolin toxicity, Cry1Ab cytotoxic effects were potentialised when sensitive cells were pre-treated with forskolin (Zhang et al., 2006). In light of these observations, they proposed that if cAMP is a key player in toxin effect through activation of PKA, specific PKA-dependent effector(s) mediating downstream cell death activity might be stimulated by Cry1Ab but not by forskolin. All these observations raise the question of how specificity is maintained in this cAMP/PKA system, activated by both forskolin and toxins (Cry1Ab and Cry1Ca) but where only Bt toxins are capable of generating cellular events leading to cell death. Since we identified Ca^2+^ as another important molecule involved in the Cry1Ca mode of action, we might think that through toxin pore formation, Ca^2+^ entry in the cell would stimulates Ca^2+^-dependent events interacting with the PKA pathway. The protein kinase C (PKC) pathway could be such Ca^2+^-dependent event which, together with the PKA activation, would allow the toxin to perform its cytotoxic effect. PKC is a large superfamily of protein kinases comprising numerous members activated by elevation of intracellular Ca^2+^ concentration (Webb et al., 2000). Coordinated activity and cross-talk between the PKA and PKC pathways have been shown in multiple cellular processes (Robinson-White and Stratakis, 2002). It might be possible that such a scenario was encountered in our intoxicated cells where forskolin was not sufficient, and Ca^2+^ was necessary, to promote cell death using Cry1Ca. It is worth noting that since the intracellular responses to Cry1 toxins studied using cell lines in culture remain controversial, this scenario will have to be validated in larvae of target insects. Finally, we observed in both selected resistant cell lines the upregulation of a gene described as the homologous of *S. exigua* activated C kinase receptor (RACK1). This PKC-activated binding protein (Mochly-Rosen, 1995) has been shown as a specific binding partner of a cAMP-phosphodiesterase (PDE) and was proposed as a potential point of cross-talk between the cAMP and PKC signalling pathways (Bird et al., 2010; Yarwood et al., 1999). RACK1 and its partners could, after intoxication by Cry1Ca, be the node of convergence of the two signalling pathways mentioned above.

In summary, Sf9-LD_80_ cells, which were the most resistant to Cry1Ca toxicity, were affected in cAMP production. Furthermore, our results have shown the importance of Ca^2+^ and Mg^2+^ ions in the toxicity of Cry1Ca. These ions may participate in different ways to the establishment of a cAMP-dependent signalling pathway following the binding of the toxin to the membrane of Sf9 cells. Both the signalling model and the pore forming model may contribute to Cry1Ca toxicity as was observed in the third model of toxin action proposed by Jurat-Fuentes and colleagues for Cry1A toxin, or more recently for the pore-forming α-Toxin from *Clostridium septicum* (Chakravorty et al., 2015; Jurat-Fuentes and Adang 2006). At low concentrations, Cry1Ca insertion in the plasma membrane could create pores, allowing Ca^2+^ entry in the cell and resulting in the activation of a cAMP-dependent cell death pathway. Future studies focusing on the identification and characterization of proteins involved in cAMP turnover, such as PDEs and their regulators, may identify new resistance factors, and contribute to a better understanding of the molecular mode of action of Cry1Ca and insect resistance mechanisms.

## MATERIALS AND METHODS

### Insect cell lines culture and selection

Sf9 cell lines (ATCC^®^ Number: CRL-1711™; Invitrogen) from ovarian tissue of pupal stage lepidopteran *S.* *frugiperda* were routinely maintained in complete medium (Grace's Insect Medium, Cambrex) containing 10% foetal bovine serum (FBS), penicillin (50 Ul/ml) and streptomycin (50 μg/ml), at 28°C, and spread when 80% confluence was reached. For resistant strain selection, cells were plated onto six-well plates until they reached 80% confluence and treated for 24 h at 28°C, either with Cry1Ca toxin at the lethal dose giving 50% dead cells (LD_50_), or at the lethal dose giving 80% dead cells (LD_80_). These lethal doses were calculated for Sf9wt when treated with various concentrations of Cry1Ca toxin (see below). Treated cells were further rinsed with Dulbecco's phosphate buffer saline (D-PBS) and allowed to recover for 2 days. After this recovery time, cells were harvested and spread in a new six-well plate for another round of selection. Forty rounds were done using the LD_50_ or LD_80_ toxin doses. Recovered resistant cells after the selection program were referred to as Sf9-LD_50_ and Sf9-LD_80_. Potential effects of aging were evaluated on cells grown for 40 rounds without toxin (Sf9p40) as compared to eight rounds (Sf9p8). S2 cells (R690-07, Invitrogen) derived from primary culture of late stage *Drosophila melanogaster* embryos were cultured at 24°C on Schneider medium (Invitrogen).

### Cry1 toxin production and purification

Cry1Ab or Cry1Ca toxins were independently produced from Bt strain 407-(minus)pHT320 transformed with plasmid expressing the full sequence of the *cry1Ca* gene (Sanchis et al., 1999) or expressing the full sequence of the *cry*1Ab gene (gift from M. Gohar, Micalis Institute, INRA, France). Cells were grown for 65 h at 30°C in HCT media containing 0.3% glucose and 10 mg/l erythromycin, spun down by centrifugation at 6500 ***g*** for 15 min at 4°C and were passed through a Potter homogenizer. After five cycles of centrifugation/homogenisation, Cry inclusion bodies were loaded onto a discontinuous sucrose gradient (weight/volume: 79/72/67%) which was then centrifuged at 23,000 rpm for 19 h at 4°C in a SW27 rotor (Beckman). Cry inclusion bodies were collected at the interface, washed with distilled water and solubilized 1 h at 37°C in 50 mM Na_2_CO_3_/NaHCO_3_, 10 mM DTT pH 10.2. Cry concentration was determined using the Bradford method (Protein Assay Reagent, BioRad) and toxin activation was further performed via a 16 h incubation at 37°C under gentle agitation in 2% trypsin (weight/weight). Activated Cry were purified by size-exclusion chromatography on Äkta-FPLC (GE Healthcare).

### Viability test

1.5×10^5^ cells were plated per well onto a 96-well microassay plate (BD Falcon) and left for 24 h at 28°C. Then cells were incubated for 18 h in complete media containing increasing concentration of activated Cry1Ab or Cry1Ca toxins (from 10^−1^ to 10^−5^ g/l). Before addition of the toxins, cells were pre-incubated 30 min in fresh media or, when divalent ions effects were measured, in fresh media containing 5 mM divalent ion chelating agents; ethylene-diamine-tetra-acetic-acid (EDTA) or ethylene-glycol-bis(2-aminoethylether)-N,N,N,N′-tetra-acetic-acid (EGTA) (Sigma-Aldrich). After toxin incubation, cells were rapidly incubated with SYBR GoldTM dye (Molecular Probes) at a 1:10,000 dilution and observed using an inverted epifluorescence equipped microscope (Eclipse TE2000-U, Nikon) coupled to a CCD camera (Orca ER, Hamamatsu). Two pictures per toxin concentration were used to count both stained and unstained cells (300–400 total cells). Cell viability was calculated by the formula: (stained cells/total cells)×100. Data were then treated on the SigmaPlot 10.0 software (Systat Software) and typical dose-response sigmoid curves were obtained with non-linear estimation. Cry1Ca lethal doses giving 50% (LD_50_) or 80% (LD_80_) dead cells were directly deduced from the curves. Resistance ratio (RR) due to treatments were obtained by the formula: LD_50 (Sf9+treatment)_/LD_50 (Sf9 in free media)_.

### Microarray experimental design

Oligonucleotides were designed from 79148 ESTs sequences recovered from eight *S. frugiperda* tissues. The assembly analysis programme CAP3 identified 10092 contigs and singleton from these ESTs. The *S. frugiperda* microarray consisted of 9773 60-mer oligonucleotides (synthesized by Sigma-Aldrich) designed to match unique contigs or singletons and to suit hybridization conditions (average GC content: 46%; average T_m_: 46°C). The experimental design of each comparison (Sf9-LD_50_ versus Sf9p40, Sf9-LD_80_ versus Sf9p40, Sf9p8 versus Sf9p40) was made using Cy3- and Cy5-labelled cDNA. Each comparison consisted of six microarrays, three biological replicates hybridized with dye swap (fully balanced dye swap design) and duplicate spots. The microarray were hybridized with cDNA prepared as described earlier (Le Goff et al., 2006) and scanned using GenePixPro scanner (Axon,version 3.01). Experimental data and associated microarray designs have been deposited in the NCBI Gene Expression Omnibus (accession number: GSE20507, http://www.ncbi.nlm.nih.gov/geo/) using Mediante database for data transfer (Le Brigand and Barbry, 2007).

### Microarray data analysis

Data analysis was performed using the Bioconductor suite of statistical packages and *limmaGUI* (Gentleman et al., 2004; Wettenhall and Smyth, 2004). Specific expression intensity was obtained by subtracting the background intensity from the foreground intensity for each non-flag spot (all flagged spots were eliminated). The expression data were normalised by within-array normalization with the ‘loess method’ and between-array normalization using the ‘quantile method’ (Yang et al., 2003). The linear model for the series of arrays and empirical Bayes method was then applied for assessing differential expression (Smyth, 2004). The false discovery rate of the *P*-value for multiple tests was controlled using the Benjamini-Hochberg method. Differentially expressed genes were selected if the absolute value of log2-fold-change was greater than 1 and adjusted *P*-value below 0.01 and if the average intensity was greater than twice the average background. In order to provide an overall measure of evidence of differential expression, we used the Fisher's method for combining adjusted *P*-values from independent tests of significance of duplicate spots (Hess and Iyer, 2007). Lists of genes differentially up- or downregulated specifically in Sf9-LD_50_, specifically in Sf9-LD_80_ or common to both cell lines are available in the Supplementary information (Table S1).

### Immunocytochemistry and live imaging

Cells were incubated for 40 min with complete media containing 3 mg/l of activated Cry1Ca toxin. Then cells were washed with D-PBS and fixed for 20 min in 4% paraformaldehyde/PBS solution at room temperature. After two rinses in D-PBS cells were quickly rinsed in 100% methanol and incubated in PBS containing 100 mM glycine. Cells were further incubated 2 h at room temperature with gentle agitation in PBS 10% normal goat serum. Immunostainings were performed with primary antibodies to Cry1Ca (rat serum obtained from an immunization program, Eurogentec, Belgium) at the 1:2000 dilution and secondary antibodies goat anti-rat IgG, Alexa Fluor 488 conjugated (Molecular Probes). Immunostaining without primary antibodies was performed as control. Cells were then rinsed with PBS and mounted under Mowiol® 4-88 based media (Polysciences). Images were obtained using an inverted epifluorescence equipped microscope (Eclipse TE2000-U, Nikon) coupled to a CCD camera (Orca ER, Hamamatsu). Four images per experiment were used to count both stained and unstained cells (150–200 total cells) and the percentage of stained cells was calculated by the formula: (stained cells/total cells)×100. Cell morphology of living Sf9 cells was examined directly in culture wells under light microscopy after 18 h incubation in complete media containing 5 mg/l of Cry1Ca with or without 5 mM of chelator agents.

### cAMP measurement

Cells were plated onto a 24-well microassay plate (BD Falcon) until reaching 80% confluency, washed with D-PBS and incubated for 20 min at 28°C with complete media only (control, CT) or containing Cry1Ca toxin (1 mg/l) or forskolin (100 µM). Cells were further incubated 10 min in HCl 0.1 M at 4°C, scrapped, vigorously shaken and centrifuged at 600×***g*** for 10 min at 4°C. Supernatants were collected and proteins were quantified by the Bradford method assay. Amounts of cAMP/mg of protein were measured in HCl extracts using the Direct cAMP enzyme immunoassay kit according to the manufacturer instruction (Sigma-Aldrich). The cAMP fold increases were calculated by the ratio of cAMP amount in stimulated conditions divided by the cAMP amount in non stimulated cells (−).

## Supplementary Material

Supplementary information
