# Supplementary material for: Transcriptomic analysis of Spodoptera frugiperda Sf9 cells resistant to Bacillus thuringiensis Cry1Ca toxin reveals that extracellular Ca2+, Mg2+ and production of cAMP are involved in toxicity
Source: Biol Open. 2019 Mar 29;8(4):bio037085. doi: 10.1242/bio.037085 (PMC6503997; doi:10.1242/bio.037085)
Supplement: Supplementary information [file biolopen-8-037085-s1.pdf]

Supplementary information

Table S1: lists of genes differentially up- or down-regulated, with an absolute value of log2-fold-change greater than 1, specifically in Sf9-LD_{50} (sheet #1), specifically in Sf9-LD_{80} (sheet #2) or common to both resistant cell lines (sheet #3). Genes are ordered using the fold change of expression from the most up-regulated to the most down-regulated.

Click here to Download Table S1
